# Methodology for the assessment of brucellosis management practices and its vaccination campaign: example in two Argentine districts

**DOI:** 10.1186/s12917-017-1201-6

**Published:** 2017-09-07

**Authors:** M.N. Aznar, M. Arregui, M.F. Humblet, L.E. Samartino, C. Saegerman

**Affiliations:** 1 Instituto de Patobiología, INTA, CICVyA, Hurlingham, PC 1688 Buenos Aires, Argentina; 2 Research Unit of Epidemiology and Risk Analysis applied to veterinary sciences (UREAR-ULg), Center for Fundamental and Applied Research for Animal and Health (FARAH), Faculty of Veterinary Medicine, University of Liege, PC 4000 Liege, Belgium; 3 Department for Occupational Safety and Health, Biosafety and Biosecurity Unit, University of Liege, PC 4000 Liege, Belgium

**Keywords:** Bovine brucellosis, Vaccination campaign, Management practices, Immunization coverage, Argentina

## Abstract

**Background:**

In Argentina, vaccination with *Brucella abortus* Strain 19 vaccine is mandatory. The objective of the study was to develop and test a method for evaluating, in an innovative way, some farmers’ and veterinarians’ management practices in relation to brucellosis and to assess the vaccination campaign and coverage. The work took place in Brandsen and Navarro districts. Four questionnaires were designed (for officials from Local Sanitary Entities, vaccinators, vet practitioners and farmers). Responses were coded as “ideal” (0) and “not ideal” (1). To assess the relative weight of each question (“item”), experts ranked the items according to their impact on management practices and vaccination. A weighted score was then calculated. A higher weighted score was assigned to the worse practices. Farmers obtaining a global weighted score above the third quartile were classified as “inappropriately managed farms”, to be compared per type of production system and district. To assess the immunization coverage, female calves were sampled 30 to 50 days post vaccination; they were expected to react positively to serological diagnostic tests (DT+).

**Results:**

There were significantly more inappropriately managed farms and higher global scores among beef farmers and in Brandsen. Eighty three percent (83%) of female calves were DT+, significantly under the ideal immunization coverage (95%). Only 48% of farms were considered well vaccinated. DT+ results were positively associated with the Brandsen district (OR = 25.94 [4.60–1146.21] and with the farms having more than 200 cow heads ((OR = 78.34 [4.09–1500.00]). On the contrary, DT+ were less associated with vaccinators being veterinary practitioners (OR = 0.07 [0.006–0.78]). Farmers are well advised by their veterinary practitioners but they should improve some management practices.

**Conclusions:**

The vaccination campaign is globally well implemented, but the immunization coverage and some vaccinators’ practices should be improved.

This study leads to a better understanding of the most common used management and control practices regarding brucellosis, which affect its epidemiology. Any vaccination campaign should be periodically assessed to highlight possible fails. The described methodology can be extrapolated to other countries and different contexts.

## Background

Brucellosis is one of the most widespread zoonoses throughout the world. It is caused by various bacteria from the genus *Brucella* which mainly affects cattle, goats, pigs, sheep and also some other species. It leads to abortion, later permanently reduced fertility, and chronically lowered milk yields in affected animals. It can be transmitted to people via direct contact with livestock or through consuming unpasteurized milk and dairy products from an infected animal.

In cattle, brucellosis is predominantly caused by *Brucella abortus*; it is usually detected in aborting females [1] which may remain infected for their entire life. Prevalence of animal infections determines the incidence of human cases [2]. Therefore, the elimination of brucellosis from the animal compartments results in a substantially reduced incidence of the disease in humans [2, 3].

In cattle, the vaccination with *Brucella abortus* Strain 19 and RB 51 is the most successful method for preventing and controlling the infection, whereas eradication can only be achieved by applying the “test and slaughter” strategy, combined with effective preventive measures such as biosecurity and control of animal movements [2]. The implementation of an effective surveillance system, with an adequate laboratory support, is one of the crucial factors to achieve eradication [4].

In Argentina, bovine brucellosis is endemic. Up to the year 2002, most cattle isolates corresponded to *Brucella abortus* biovar 1 (epitope A dominant); however, *Brucella abortus* biovar 2 (epitope A dominant) was also reported [5]. The predominant biovar has remained stable in the country so far [6].

The disease is more controlled in dairy farms than in beef holdings because milk coming from holdings certified as Officially Brucellosis-Free is better valued by the industry. On the contrary, no compensation is provided to non-there is an unnecessary space Officially Brucellosis-Free beef farms [6]. Approximately 60% of the 6500 dairy farms are Officially Brucellosis-Free. Regarding beef, 12% (95% CI: 10% - 14%) of the 180,000 farms and 0.8% (95% CI: 0.5% - 1.05%) of the 46,000,000 cattle are infected (SENASA, unpublished data). Between 6 and 7 million of female calves are mandatorily vaccinated with Strain 19 every year.

Some methodology for assessing the farmers’ and veterinarians’ behavior concerning the disease and for evaluating its vaccination campaign should be developed to improve the surveillance system. To our knowledge, there are not many studies suggesting any methodology regarding this issue.

Therefore, the objective of this study was to develop and test a method for evaluating, in an innovative way, some farmers’ and veterinarians’ management practices in relation to brucellosis and for assessing the vaccination campaign and coverage in order to detect fails of the National Plan for the Control and Eradication of Bovine Brucellosis. We worked in two districts of the Buenos Aires province, Brandsen and Navarro.

## Methods

### National Plan for the control and eradication of bovine brucellosis in Argentina

The National Plan for the Control and Eradication of Bovine Brucellosis establishes the individual identification and mandatory sub-cutaneous vaccination of all 3 to 8 month-old females with 15–30 × 10^9^ viable *Brucella abortus* Strain 19 in 2 ml-doses (SENASA, 2002). The vaccine must be controlled and approved by the National Service for Agrifood Health and Quality (SENASA). Every vaccinated female must be permanently identified (ear tags are commonly used).

SENASA entrusts the implementation of the vaccination campaign to 310 Local Sanitary Entities (LSEs). The LSEs are institutions gathering representatives of farmers and animal health institutions (including veterinarians from SENASA and other institutions playing a local role). Each district implements its own local vaccination plan, according to the SENASA general strategy, which is thus adjusted to the different regional realities. The LSEs are responsible for planning, implementing and assessing local vaccination campaigns, which must comply with the SENASA set of rules, and target a large immunization coverage within short periods of time. To achieve that goal, LSEs vaccinators (either vet practitioners or non-veterinarians) are registered and trained by the SENASA. Each entity, under the supervision of a SENASA veterinarian, manages the vaccine supply at a local level. In most cases, foot-and-mouth disease and brucellosis vaccines are administered together. The vaccines are paid by the farmers [6]. The SENASA controls the entire immunization process.

### Area of study and selection of participants

**Fig. 1:**
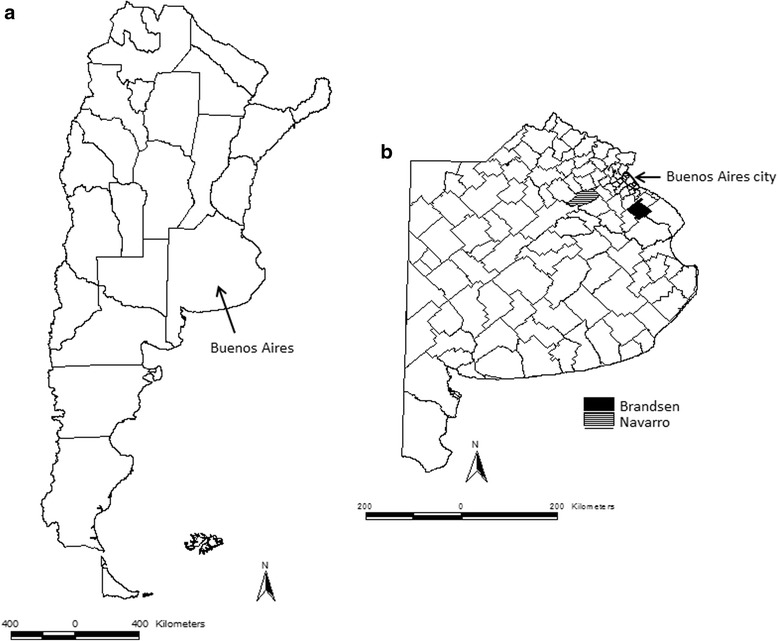
Maps showing the boundaries of Argentina and its provinces (on the left) (**a**), as well as Brandsen and Navarro districts (study area), in the province of Buenos Aires (on the right) (**b**)

This cross-sectional descriptive study covered two districts in the province of Buenos Aires, Argentina: Brandsen and Navarro (Fig. 1). Brandsen has a total of 625 bovine farms and Navarro has 648 farms (SENASA, personal communication). These districts were selected because they represent well the type of bovine production in the province and because people in charge of LSEs agreed to collaborate in the implementation of the field work. Four different types of actors (LSEs officials, vaccinators, vet practitioners and farmers) who play a key role in the immunization campaign and/or the management practices were randomly selected to be interviewed face-to-face by the corresponding author (*n* = 142). There was not a statistical design for computing the number of responses to be part of the study. However, 10% of farmers were expected to be interviewed. Answers were obtained upon grounds of convenience.

### Questionnaires design

A questionnaire for each type of actor was designed. Questionnaires had between 6 and 18 questions (“items”) with closed dichotomous answers (Tables 1, 2, 3 and 4). Possible answers for each item were coded as “ideal” (code = 0) and “not ideal” (code = 1). Besides, vet practitioners’ personal opinions on vaccination aspects were recorded.

**Table 1:** Farmers’ questionnaire

Items	Answers		Experts’ opinion		Weighting
	Ideal	Not ideal	Average [A]	CV	Weighted Factor [B]
Identification of all vaccinated females	Yes	No	6.4	0.699	0.064
Identification of females unvaccinated due to their age (< 3 months)	Yes	No	3.4	0.609	0.034
Coverage per farm	95%–100%	<95%	16.4	0.372	0.164
Existence of reproductive records in the farm	Yes	No	4.0	0.306	0.040
Diagnostic of abortions, placental retentions or no-pregnancy	Yes	No	8.8	0.478	0.088
Isolation of animals with reproductive disorders consistent with brucellosis	Yes	No	10.2	0.497	0.102
Sale of animals with clinical signs of brucellosis	Yes	No	12.0	0.343	0.120
Destination of the animals sold with clinical signs of brucellosis	Slaughterhouse	Other destination	3.6	1.168	0.036
Purchase of cattle from Officially Brucellosis-Free farms	Yes	No	11.0	0.497	0.110
At purchase: brucellosis serological testing of females >18 months and males 6 months	Yes	No	11.0	0.203	0.110
Isolation of purchased cattle	Yes	No	5.6	0.763	0.056
Isolation of calving cows	Yes	No	5.6	0.508	0.056
Isolation of primiparous cows	Yes	No	2.0	1.514	0.020

Legend: *CV* = coefficient of variation [A] Average of points distributed by the experts, per item. [B] Weighted factor per item. This value was obtained after dividing [A] by the total of points distributed by each expert (100 points). The sum of all weighted items equals 1

List of items, possible answers, experts’ opinion and weighting

**Table 2:** Vet practitioners’ questionnaire

Items	Answers		Experts’ opinion		Weighting
	Ideal	Not ideal	Average [A]	CV	Weighted Factor [B]
The veterinarian suggests a brucellosis serological diagnostic test in case of abortions	Yes	No	105.0	0.199	0.210
The veterinarian suggests a brucellosis serological test in case of retained placenta, no-pregnancy or calf/calves born weak	Yes	No	100.0	0.176	0.200
The veterinarian suggests a brucellosis serological test in case of orchitis or epididymitis in males	Yes	No	63.0	0.407	0.126
The veterinarian suggests quarantine for purchased cattle	Yes	No	77.0	0.374	0.154
The veterinarian suggests a brucellosis serological test when purchasing females >18 months and males >6 months	Yes	No	120.0	0.093	0.240
The veterinarian performs a brucellosis serological test before selling cattle for reproductive reasons	Yes	No	35.0	0.391	0.070

Legend: *CV* = coefficient of variation [A] Average of points distributed by the experts, per item. [B] Weighted factor per item. This value was obtained after dividing [A] by the total of points distributed by each expert (100 points). The sum of all weighted items equals 1

List of items, possible answers, experts’ opinion and weighting

**Table 3:** Vaccinators’ questionnaire

Items	Answers		Experts’ opinion		Weighting
	Ideal	Not ideal	Total [A]	CV	Weighted Factor [B]
Checking of the vaccine expiration date before its use	Yes	No	38.0	0.547	0.076
A different syringe is used for Brucellosis and foot-and-mouth vaccines	Yes	No	31.0	0.447	0.062
There must be vacuum in the flask containing the lyophilized product	Yes	No	36.0	0.432	0.072
Softly shake of the vaccine flask after adding the diluent to the lyophilized product	Yes	No	34.0	0.351	0.068
Make sure there is no air in the syringe before injecting the vaccine	Yes	No	29.0	0.446	0.058
Frequency at which air verification is performed	Each time it is uploaded	Other	17.0	0.675	0.034
The needle is changed between farms	Yes	No	38.0	0.799	0.076
Syringe calibration	Yes	No	41.0	0.264	0.082
Frequency of syringe calibration	Each time it is uploaded	Other	21.0	0.547	0.042
Homogenization of the vaccine flask during vaccination	Yes	No	39.0	0.246	0.078
Frequency of vaccine homogenization	Each time it is uploaded	Other	16.0	0.978	0.032
Maximal delay for using the vaccine after adding the diluent to the lyophilized product	<5 h	Other	42.0	0.246	0.084
Vaccine storage place while vaccinating	In a cooled box, in the shade	Other	35.0	0.335	0.070
The vaccine is injected again if some of it drops after the first injection	Yes	No	23.0	0.6974	0.046
Vaccinators notice the double injections in the vaccination records	Yes	No	7.0	0.719	0.014
Future of empty vaccine flasks once vaccination is completed	LSEs	Other	10.0	0.692	0.020
The number of vaccinated females and doses used are recorded	n vaccinated females and doses used	Other	32.0	0.422	0.064
Vaccinators wear personal protective equipment (e.g. gloves, goggles, and overalls)	Yes	No	11.0	0.956	0.022

Legend: *CV* = coefficient of variation [A] Average of points distributed by the experts, per item. [B] Weighted factor per item. This value was obtained after dividing [A] by the total of points distributed by each expert (100 points). The sum of all weighted items equals 1

List of items, possible answers, experts’ opinion and weighting

**Table 4:** Questionnaire for Local Sanitary Entities (LSE) officials

Items			Experts’ opinion		Weighting
	Ideal	Not ideal	Total [A]	CV	Weighted Factor [B]
The LSE audits the vaccination process	Yes	No	51.0	0.107	0.102
Frequency of LSE audits	Twice per campaign	Other	35.0	0.484	0.070
Aspects audited by the LSE	All of them	None of them	37.0	0.377	0.074
The LSE advertises dates of the vaccination campaign beforehand	Yes	No	30.0	0.440	0.060
The LSE plans the date for vaccination with the farmers beforehand	Yes	No	36.0	0.432	0.072
The LSE controls the temperature at which the vaccine is supplied by the producing laboratories	Yes	No	50.0	0.353	0.100
Temperature at which the vaccine is stored at the LSE	2–8 ° C	Other	55.0	0.203	0.110
The LSE checks the temperature of the fridge in which the vaccines are stored	Yes	No	57.0	0.192	0.114
Frequency of temperature control	Twice a day	Other	37.0	0.454	0.074
The LSE supplies the vaccine to vaccinators in cooled boxes	Yes	No	55.0	0.240	0.110
The LSE receives the surplus of vaccine brought back by vaccinators by the end of the day	Yes	No	22.0	0.565	0.044
The LSE controls the temperature at which the vaccine surpluses are brought	Yes	No	35.0	0.404	0.070

Legend: *CV* = coefficient of variation [A] Average of points distributed by the experts, per item. [B] Weighted factor per item. This value was obtained after dividing [A] by the total of points distributed by each expert (100 points). The sum of all weighted items equals 1

List of items, possible answers, experts’ opinion and weighting

### Global weighted scores

To assess the relative weight of each item, a panel of Argentine experts (*n* = 5) in the field of brucellosis and Sanitary Policies were consulted. They were asked to distribute a total of 100 points among the items under study, for each type of actor, on the basis of the impact they can have on the brucellosis managing practices and effectiveness of the vaccination campaign. The parameter with major impact received the maximum of points. The average of points allocated by the experts to each item was divided by the total of points (*n* = 100) to obtain a weighted factor. Then, a weighted score was calculated for each item through multiplying the code (1 or 0) by the weighting factor. A higher weighted score was related to worse managing and vaccinating practices. A global weighted score was obtained by adding up each weighted score per respondent.

Farmers obtaining a global weighted score above the third quartile were classified as “inappropriately-managed farms”, since the authors fixed a threshold of 75% of ideal answers for the appropriate managed farms. The distribution of inappropriately-managed farms was compared per type of production (dairy vs. beef) and district (Brandsen vs. Navarro).

Some questions were used to compare both districts and production systems, but no comparison with the ideal answers (based on experts’ opinion) was carried out.

### Immunization coverage

To evaluate the compliance of vaccination field practices with SENASA regulations, serum samples of a subgroup of the farms were randomly taken from female calves, 30 to 50 days post vaccination (DPV), during the 2014-autumn brucellosis vaccination campaign. Sample size for number of female calves per farm was computed assuming a 10% relative error with a 95% confidence interval and a 95% of vaccinated cattle, which would be expected in a mandatory immunization campaign. A total of 20 female calves per farm (or all of them if less than 20 calves were vaccinated) fulfilled the previous assumptions and were randomly sampled per farm. The Buffered Plate Antigen Test and the Complement Fixation Test were the diagnostic tests (DT) selected to validate the exposure to vaccine.

Any female calf vaccinated 30 to 50 days before the sampling date with the commercial brucellosis vaccine approved by SENASA (2 ml-dose) was expected to react positively to the DT (DT+ = Buffered Plate Antigen Test + and/or Complement Fixation Test +) [7, 8]. We classified a farm as “well vaccinated” if the proportion of DT+ was not significantly lower than the ideal 95%. DPV on which female calves were sampled were categorized as follows: 30–35 and 35–50.

### Statistical analyses

The comparison of inappropriately-managed farms and of each item per district or production system was carried out with the Fisher’s exact test. The comparisons of weighted scores per respondent were performed with the Wilcoxon Rank Sum Test.

A univariate, and secondly a multivariate logistic regression using backward stepwise analysis, were applied to assess the association between the dependent “DT+/DT-” and independent variables [9]. Independent variables were categorized as dichotomous: vaccinator (vet practitioner/non-veterinarian), productive system (dairy/beef), age of female calves at the time of vaccination (4–6 months/7–8 months), individual identification of female calves (identified or not), DPV for heifer sampling (30–35 DPV/35–50 DPV), herd size (lower or higher than 200 cows, which is the median of cows per farm in both districts farms). Variables showing a significant univariate test at an initial *p* value < 0.2 were included in a multivariate model. In addition, a backward elimination of variables allowed assessing collinearity [10]. Any variable inducing a > 20%-variation of odds ratio (OR) was included in the final model [11]. In that final model, all pairwise interactions between variables, if biologically relevant, were examined for significance. Goodness-of-fit was assessed using the Hosmer-Lemeshow test.

## Results

Responses from 113 farmers (approximately 10% of farmers of both districts, 55 from dairy and 58 from beef farms), 11 private veterinarians (which represent 80% of the veterinarians), 16 vaccinators (all of them) and the 2 LSEs officials, were collected during the years 2013 and 2014. A total of 52 (46%) surveyed farms were Officially Brucellosis-Free, out of which 12 (23%) were beef holdings.

### Farmers and private vet practitioners

Almost all interviewed farmers (96%; *n* = 108) were advised by a private vet practitioner. Dairy and Officially Brucellosis-Free farmers kept significantly more reproductive records and tried significantly (*P* < 0.05) more often to diagnose causes of abortions and reproductive disorders than beef and non-Officially Brucellosis-Free farms, respectively. Although individual identification of vaccinated females is mandatory, beef farms identified significantly less animals (P < 0.05) than dairy holdings. The proportion of reported abortions was significantly (*P* < 0.05) higher in dairy farms. Only 14% (*n* = 16) of farmers isolated cattle with reproductive disorders consistent with brucellosis; the destination of all sold animals was the slaughterhouse. Regarding isolation of cattle suspected of brucellosis, no significant difference was observed between dairy and beef holdings on one hand, and between Officially Brucellosis-Free and non-Officially Brucellosis-Free certified farms, on the other hand (*P* > 0.05). Concerning purchase, neither the production system, nor the disease status showed a significant effect: out of 48 (42%) farmers who purchased cattle in the previous year, 18 (38%) did it from farms certified as Officially Brucellosis-Free; 13 of them (27%) resorted to a serologic diagnostic test before introducing animals into their herd (although suggested by all vet practitioners), and only 4 farmers (8%) isolated purchased cattle (although suggested by 7 veterinarians [63%]). Regarding immunization coverage of farms, 13% of farmers admitted that not 100% of females were vaccinated; such observation concerned mostly beef farms (*P* < 0.05), reporting no mating season. Calving cows were isolated from the herd by 47% of farmers (*n* = 53), and significantly (*P* < 0.05) more frequently in dairy farms. Forty-five percent (45%) of farmers (*n* = 14 dairy and 10 beef farms) isolated females at first calving.

At least one case of bovine brucellosis was reported by 4 vet practitioners (63%) for the previous year. All vet practitioners (*n* = 11) suggested to test serologically the females which aborted or showed reproductive disorders (e.g. retained placenta, no-pregnancy or delivering weak calves); 9 vets (81%) suggested serology for males with orchitis and 4 of them (36%) for epididymitis. In case of bovine brucellosis, the investigation in other domestic species (especially swine) was implemented by 6 vets (54%).

Regarding personal opinion of vet practitioners on immunization: (i) individual identification is poor, (ii) immunization coverage is incomplete, particularly in beef farms with no mating season (individual identification is not the rule, and age of calves is not reliable), (iii) vaccination is difficult to perform due to poor conditions of alleys, (iv) a 5 ml-dose would be better than a 2 ml-dose (indeed, only 1 ml is injected if the syringe is not correctly calibrated), (v) vaccine has been useful in reducing brucellosis prevalence (even in holdings where no other control measures have been implemented) and (vi) most farmers are used to vaccinate, even if it is a financial charge (few unvaccinated females are reported, only among farmers who do not immunize females under 3 months of age).

### Vaccinators

In both districts, vaccinators were either vet practitioners or non-veterinarians. All of them are used to check the vaccine expiration date, the vacuum in the vaccine flask prior to dilution and to store the vaccine in coolers, in the shade, while vaccinating. Six vaccinators (38%) calibrated the syringe at the beginning of the vaccination day, 4 of them (25%) each time that they filled it, 2 of them (12%) at the beginning and each time that the vaccine is filled; 4 vaccinators (25%) never calibrate the syringe. Vaccinators (100%) do not immunize females that look older than 8 months of age, so as not to interfere with diagnostic tests. Half of them homogenize the vaccine during immunization. In case of vaccine dropping after injection, 12 vaccinators (75%) inject it again, but only 6% (*n* = 10) record the double injection in the vaccination certificate.

### LSEs officials

LSEs officials audited vaccinators’ performance at least once per vaccination campaign on the following topics: high immunization coverage in farms, good vaccination practices, and animal welfare.

At the LSEs, the temperature of refrigerators where vaccine flasks are stored were checked twice a day. Vaccine supply by the producing laboratories and its distribution to vaccinators in coolers were also assessed.

### Comparison of practices between respondents and experts

#### Farmers

The 5 experts were consistent in assuming that the following items were the most susceptible to impact farmers’ good management practices (low coefficient of variation [CV], as shown in Table 1): sales of cattle suspected of brucellosis, good immunization coverage per farm, purchase of cattle from Officially Brucellosis-Free holdings, and serological tests upon purchasing animals.

Considering all farmers (*n* = 113), the median global weighted score reached 0.28 [range 0–0.88; First quartile = 0.21 and Third quartile = 0.41]. For dairy farms, that score was 0.22 [range 0–0.68; First quartile = 0.16 and Third quartile = 0.31], compared to 0.36 for beef holdings [range 0.12–0.87; First quartile = 0.27 and Third quartile = 0.49]. When comparing both districts, median global weighted scores reached 0.32 [range 0–0.88; First quartile = 0.24 and Third quartile = 0.43] and 0.25 [range 0.06–0.87; First quartile = 0.16 and Third quartile = 0.39] for Brandsen and Navarro, respectively.

**Fig. 2:**
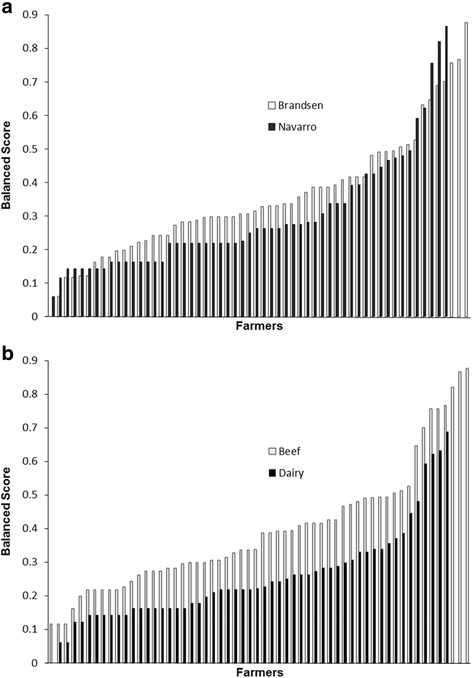
Farmers’ weighted scores in function of production system (**a**) and district (**b**). Legend: **a**: Dairy (First quartile = 0.16; median = 0.22; Third quartile = 0.31) and Beef (First quartile = 0.27; median = 0.36; Third quartile = 0.49). **b**: Brandsen (First quartile = 0.24; median = 0.32; Third quartile = 0.43) and Navarro (First quartile = 0.16; median = 0.25; Third quartile = 0.39)

Twenty-three beef farms (36.7%) and 6 dairy holdings (10.9%) fell into the ‘inappropriately-managed farms’ category (*P* < 0.05); Brandsen district accounted for 29.3% of them, while 17.2% where located in Navarro district (*P* > 0.05; n total = 58). As illustrated in Fig. 2, the global weighted scores were significantly higher (*P* < 0.05) in beef farms and in Brandsen district.

#### Veterinary practitioners

For vet practitioners, the most impacting item was to suggest serological testing for females with reproductive disorders (low CV, as shown in Table 2). The median global weighted score reached 0.13 [range 0–0.48; First quartile = 0.13 and Third quartile = 0.28] and no statistical difference was observed between both districts.

#### Vaccinators

As shown in Table 3, the 5 experts were consistent in assuming the following items to be the most susceptible to impact the vaccinators’ performance (low CV): maximal delay for using the vaccine, syringe calibration and vaccine homogenization. The median global weighted score was 0.17 [range 0.06–0.41; First quartile = 0.12 and Third quartile = 0.27].

A global weighted score above the Third quartile was obtained for 4 vaccinators (25%). No significant difference was observed between both districts. Vet practitioners obtained a higher median score compared with non-veterinarians (0.23 vs. 0.12, respectively), but that difference was not significant.

#### LSEs

Table 4 summarizes the items included in the questionnaires submitted to LSEs officials: experts agreed that vaccine storage at an appropriate temperature, as well as audits, had the highest impact (lowest CV). The global weighted scores were 0.102 for Brandsen and 0.114 for Navarro.

#### Vaccination coverage

A total of 393 female calves (262 from Brandsen and 131 from Navarro) from 21 farms (5 dairy and 9 breeding farms from Brandsen, 3 dairy farms and 4 breeding from Navarro) were sampled. Table 5 summarizes the number of sampled farms, the number of well vaccinated and the proportion of well vaccinated per DPV category, as well as individual results of diagnostic testing. Female calves were correctly identified in 52% of farms (*n* = 11). A DT+ result was registered for 83% (*n* = 328) of sampled female calves which was significantly lower compared to an ideal 95%-immunization coverage (*P* < 0.05). Also, among all DPV categories, the proportion of well vaccinated farms was significantly lower compared to an ideal 95%-immunization coverage (*P* < 0.05).

**Table 5:** Immunization coverage at farm and individual levels

A) At farm level			
DPV	nf	n WV	% WV
30–35	8	4	50^a^
35–50	13	6	46^a^
Total	21	10	48^a^
B) At individual level			
DPV	nfc	n DT+	% DT+
30–35	143	127	89
35–50	250	201	80^a^
Total	393	328	83^a^

Legend: *DPV* = days post vaccination; nf = number of farms; nWV = number of “well vaccinated” farms (farms where the proportion of DT+ was not significantly lower compared to an ideal 95%-immunization coverage); %WV = percentage of “well vaccinated” farms; nfc = number of sampled female calves; n DT + = number of female calves positive to the diagnostic tests; % DT = percentage of female calves positive to the diagnostic tests; ^a^significant differences with the ideal 95%-coverage

At least one heifer was DT+ in each farm, and all tested female calves were DT+ in 33% of holdings (*n* = 7). Nevertheless, 48% of farms (*n* = 10) were assumed to be well vaccinated.

Batches of vaccines used in the sampled farms were approved by SENASA and were not beyond expiration date.

Regarding variables potentially influencing a DT+ status, no significant effect was observed for DPV, production system, Officially Brucellosis-Free vs. non- Officially Brucellosis-Free status, individual identification or age at the time of vaccination. The final multivariate logistic regression included 4 independent variables: district, vaccinator type, farm size and age at the time of vaccination. A DT+ status was significantly and positively associated with Brandsen district (OR = 25.94 [4.60–1146.21] and with farms having > 200 cows (OR = 78.34 [4.09–1500.00]). On the other side, the vaccinator being a vet practitioner was less associated with a DT+ status (OR = 0.07 [0.006–0.78]). All pairwise interactions were tested. The Hosmer-Lemeshow test showed that the final model fit the data correctly (Chi^2^ = 0.26, df = 1, *P* = 0.96).

## Discussion

Although well advised by their veterinarians, farmers should improve some management practices. In particular, beef farmers should investigate abortions and reproductive disorders, and focus on individual identification of vaccinated female calves, as stated by the SENASA national regulations. Farmers should purchase cattle from Officially Brucellosis-Free farms and perform serological diagnostic tests and/or implement a quarantine procedure before introducing animals into the herd.

Vaccination is the most successful method to prevent and control brucellosis in endemic countries [4]. Increasing the immunization coverage among females is essential for animal and public health, but also for the farmers’ economy [2, 3]. Besides, animal mass vaccination allows rewording money for the public health sector [12].

Although the percentage of sampled farms was small, we could identify that the vaccination campaign in the districts under study is globally well implemented, but some aspects should be improved, such as coverage and some vaccinators’ practices. In fact, the relative low coverage (significantly below the 95%-expected in a mandatory vaccination campaign) is not due to the vaccine quality, which is controlled by SENASA, but rather to poor vaccination practices.

Immunization coverage (DT+ status) was better in Brandsen district, since DT+ female calves are almost 26 times more likely to be encountered. Farms having more than 200 cows seem better vaccinated, as finding DT+ female calves is 78 times more likely. Strict records and good alleys to perform the vaccination might explain such difference. Cattle vaccinated by vet practitioners are 0.07 times less likely to be DT+, but additional studies involving larger samples are needed. Beef farms should improve their recording system. In fact, the higher proportion of reported abortions in dairy farms is probably due to better records.

This original methodology, designed to monitor tuberculosis skin testing among vet practitioners, was initially performed in Belgium and in France. A scoring scale was built, based on experts’ opinion, and a global score was calculated and compared with vet practitioners’ responses [13, 14]. The same methodology could be adapted to evaluate farmers’ and veterinarians’ management practices in relation to other diseases, in different contexts and different countries.

Regular evaluation of systematic and mass vaccination campaigns is of great importance in order to quantify their effectiveness [15], detect problems, and monitor the effect of interventions aimed at correcting them. During the years 2004, 2007, 2008 and 2011, the systematic foot-and-mouth disease mass vaccination campaigns were assessed in some districts of Argentina [16], but no study had focused on brucellosis so far. In addition, the methodology applied in those surveys did not consist in questionnaires answered by the main actors, but only relied on serological tests to estimate the cattle humoral immunity level. Regarding brucellosis, the former studies estimated the disease prevalence at country level [6]; the present study is thus innovative in evaluating brucellosis vaccination practices and coverage.

As regards farmers and vaccinators, they should be informed on how to improve their management and immunization practices, respectively. Improving auditing of the whole vaccination process should be the main objective of people in charge of vaccination campaigns.

This study has been a first approach of the evaluation of brucellosis vaccination. It should be repeated in the future with more samples to obtain more precise confidence intervals for the OR. It should be repeated not only in the same districts to assess improvements among all actors, but it could also be applied in other districts. The study also allowed awareness on the campaign through evidence-based data of serology. Nevertheless, the questionnaire and the serological surveys are complementary tools; they should be implemented simultaneously: the questionnaire details the vaccination process, per actor, while the serological survey assesses the immunization coverage. Brandsen district perfectly illustrates that recommendation, even if farmers’ practices were poor, vaccination was better implemented.

## Conclusions

In this study, we developed and tested a method to assess the farmers’ and veterinarians’ behavior concerning the disease and to evaluate the vaccination campaign. It leads to a better understanding of the most common used management and control practices regarding the disease, which may affect its epidemiology. It highlights the need for policy makers to be aware of farmers’ and veterinarians’ practices, to detect strengths and weaknesses on diseases management in order to adapt, improve and monitor Control and Eradication Plans. It is also useful to evaluate people in charge of vaccines application, as well as the operative aspects of the whole vaccination campaigns.

Any vaccination campaign should be periodically evaluated to highlight the aspects that require improvement. The herein explored methodology, and the questionnaires combined with serologic tests are interesting to appropriately assess the subject. To our knowledge, not many studies exist on the evaluation of vaccination campaigns, being this one, though, innovative. It is not only applicable to Argentina, but also to the numerous countries that use vaccines against brucellosis or other diseases.

Our work is just a pilot study which might be the basis of a more extensive study in the rest of the country and in other countries. It can be extrapolated to other countries and different contexts.

These results were presented and discussed with the SENASA authorities. Some changes and improvements were consecutively observed during the 2015 brucellosis vaccination campaign, e.g. increased trends in animal identification and the control of vaccinators while performing their task.

## Abbreviations

CV: coefficient of variation
DPV: days post vaccination
DT: diagnostic tests
LSEs: Local Sanitary Entities
OR: odds ratio
SENASA: National Service for Agrifood Health and Quality
